# DNA Methylation and Histone Modifications Regulate *De Novo* Shoot Regeneration in *Arabidopsis* by Modulating *WUSCHEL* Expression and Auxin Signaling

**DOI:** 10.1371/journal.pgen.1002243

**Published:** 2011-08-18

**Authors:** Wei Li, Hui Liu, Zhi Juan Cheng, Ying Hua Su, Hua Nan Han, Yan Zhang, Xian Sheng Zhang

**Affiliations:** State Key Laboratory of Crop Biology, Shandong Key Laboratory of Crop Biology, College of Life Sciences, Shandong Agricultural University, Taian, China; Peking University, China

## Abstract

Plants have a profound capacity to regenerate organs from differentiated somatic tissues, based on which propagating plants *in vitro* was made possible. Beside its use in biotechnology, *in vitro* shoot regeneration is also an important system to study *de novo* organogenesis. Phytohormones and transcription factor WUSCHEL (WUS) play critical roles in this process but whether and how epigenetic modifications are involved is unknown. Here, we report that epigenetic marks of DNA methylation and histone modifications regulate *de novo* shoot regeneration of *Arabidopsis* through modulating *WUS* expression and auxin signaling. First, functional loss of key epigenetic genes—including *METHYLTRANSFERASE1* (*MET1*) encoding for DNA methyltransferase, *KRYPTONITE* (*KYP*) for the histone 3 lysine 9 (H3K9) methyltransferase, *JMJ14* for the histone 3 lysine 4 (H3K4) demethylase, and *HAC1* for the histone acetyltransferase—resulted in altered *WUS* expression and developmental rates of regenerated shoots *in vitro*. Second, we showed that regulatory regions of *WUS* were developmentally regulated by both DNA methylation and histone modifications through bisulfite sequencing and chromatin immunoprecipitation. Third, DNA methylation in the regulatory regions of *WUS* was lost in the *met1* mutant, thus leading to increased *WUS* expression and its localization. Fourth, we did a genome-wide transcriptional analysis and found out that some of differentially expressed genes between wild type and *met1* were involved in signal transduction of the phytohormone auxin. We verified that the increased expression of *AUXIN RESPONSE FACTOR3 (ARF3)* in *met1* indeed was due to DNA demethylation, suggesting DNA methylation regulates *de novo* shoot regeneration by modulating auxin signaling. We propose that DNA methylation and histone modifications regulate *de novo* shoot regeneration by modulating *WUS* expression and auxin signaling. The study demonstrates that, although molecular components involved in organogenesis are divergently evolved in plants and animals, epigenetic modifications play an evolutionarily convergent role in this process.

## Author Summary

Plants have a strong ability to generate organs from differentiated somatic tissues. Due to this feature, shoot regeneration *in vitro* has been used as an important way for producing whole plants in agriculture and biotechnology. Phytohormones and the transcription factor WUSCHEL (WUS) are essential for reprogramming during *de novo* shoot regeneration. Epigenetic modifications are also critical for mammalian cell differentiation and organogenesis. Here, we show that epigenetic modifications mediate the *de novo* shoot regeneration in *Arabidopsis*. Mutations of key epigenetic genes resulted in altered *WUS* expression and developmental rates of regenerated shoots *in vitro*. Bisulfite sequencing and chromatin immunoprecipitation revealed that the regulatory regions of *WUS* were developmentally regulated by both DNA methylation and histone modifications. By transcriptome analysis, we identified that some differentially expressed genes between wild type and *met1* are involved in signal transduction of the phytohormone auxin. Our results suggest that DNA methylation and histone modifications regulate *de novo* shoot regeneration by modulating *WUS* expression and auxin signaling. The study demonstrates that, although molecular components involved in organogenesis are divergently evolved in plants and animals, epigenetic modifications play an evolutionarily convergent role during *de novo* organogenesis.

## Introduction

Differentiated somatic tissues of plants can be reprogrammed to generate various organs, a process called *de novo* organogenesis. This feature is not only critical for *in vitro* plant propagation and application of biotechnology, but also provides a good experimental system for understanding regulatory mechanisms underlying organogenesis.

Recent studies have revealed some molecular mechanisms underlying *de novo* shoot regeneration in *Arabidopsis* [1]–[4], in which WUS, a transcription factor, plays a key role [5], [6]. WUS is a master regulator of stem cell fate determination in shoot apical meristem (SAM), on which many signaling pathways converge [7]. It turned out to be also critical for *de novo* shoot regeneration. During *de novo* shoot regeneration in *Arabidopsis*, expression of *WUS* is sufficient to specify the organizing center, which is required for stem cell induction and subsequent shoot regeneration [5], [6], [8]. *WUS* induction is also essential for shoot formation during *de novo* somatic embryogenesis [9]. Induction of the *WUS* expression during *de novo* shoot regeneration was regulated by the master phytohormone auxin [2], [5]. Recently, *WUS* expression in the organizing center of the *Arabidopsis* plant SAM was shown to be regulated by epigenetic modifications [10].

Epigenetic modifications, including DNA methylation and histone modifications, occur extensively during cellular differentiation and development in mammals [11]–[13]. In mammals, the patterns of DNA methylation are established by *de novo* DNA methyltransferase 3 (DNMT3) family and maintained by methyltransferase DNMT1 [14]. DNMT1 plays a vital role in controlling the self-renewal and differentiation of stem cells during hematopoiesis and leukemogenesis and is critical for progenitor maintenance and self-renewal in mammalian somatic tissues [15], [16]. DNA methylation and histone modifications regulate gene expression through changing chromatin structure and transcriptional activities [17]–[19]. For instance, transcriptional repression is associated with hypermethylation of DNA, histone deacetylation and histone H3K9 methylation, whereas active chromatin is linked with hypomethylation of DNA, histone acetylation and histone H3K4 methylation [17], [20].

In plants, pattern changes of DNA methylation and histone modifications leading to changes in chromatin state occur in plant cells undergoing dedifferentiation [21]–[24]. Furthermore, DNA methylation at some promoters is critical for establishing or maintaining the undifferentiated cell state in plants [25]. However, whether and how epigenetic modifications are involved in cell differentiation during *de novo* shoot regeneration is unknown. Here we showed that mutations of key epigenetic genes altered *WUS* expression and developmental rates of regenerated shoots *in vitro*. In addition, epigenetic marks of DNA methylation and histone modifications in the regions of *WUS* underwent dynamic changes during *de novo* shoot regeneration, correlating with dynamic *WUS* expression levels. Genome-wide transcriptional analysis indicated that some genes involved in auxin signaling and meristem development were methylated within the callus, but were demethylated following an induction treatment. Based on these results, we propose that dynamic DNA methylation and histone modifications mediate *de novo* shoot regeneration in *Arabidopsis* through *WUS* and auxin signaling.

## Results/Discussion

### Mutations interfering with epigenetic modifications changed developmental rates of *de novo* shoot regeneration

To find out whether DNA methylation and histone modifications played roles in *de novo* shoot regeneration, we first compared the capacity and rates of shoot regeneration between wild type and various epigenetic mutants after calli being transferred onto a shoot induction medium (SIM) from a callus induction medium (CIM) [26]. *Arabidopsis METHYLTRANSFERASE1* (*MET1*), *KRYPTONITE* (*KYP*), *JMJ14* and *HISTONE ACETYLTRANSFERASE1* (*HAC1*), among diverse genes involved in epigenetic modifications, have been well characterized [27]–[31]. *MET1* is an ortholog of DNMT1, which maintains DNA methylation directly at CpG motif and indirectly at non-CG motif [27], [32], [33]. Functional loss of *MET1* resulted in delayed transition from vegetative phase to reproductive phase [32]. *KYP* encodes histone H3K9 methyltransferase, and mutation of which resulted in abnormal number of floral organs [28]. *JMJ14* encodes histone H3K4 demethylase that inhibited flowering under long-day condition [29], [34]. *HAC1* encodes histone acetyltransferase, regulating flowering time through histone acetylation [31], [35]. We used the final percentage of shoot primordia on SIM to reflect the capacity of *de novo* shoot regeneration, whereas the timely appearance of shoot primordia to reflect their developmental rates.

**Figure 1:**
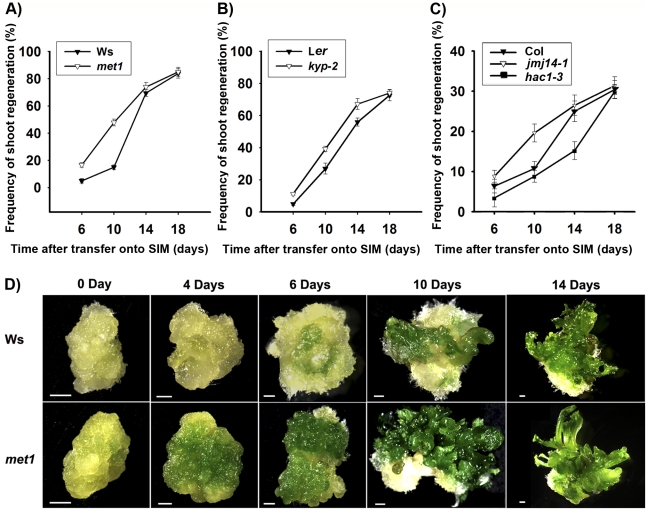
Mutation in key epigenetic genes alters the rate of *Arabidopsis* shoot regeneration *in vitro*. A) Frequency of shoot regeneration from calli of the wild type (Ws) and the mutant *met1*. B) Frequency of shoot regeneration from calli of the wild type (L*er*) and the mutant *kyp-2*. C) Frequency of shoot regeneration from calli of the wild type (Col) and the mutants *jmj14-1* and *hac1-3*. Calli were induced from pistils on CIM, and were then transferred onto SIM for shoot induction. Data are presented as mean values from three sets of biological replicates. In each replicate, at least 60 calli were examined. D) Calli of the wild type (Ws) and the mutant *met1* cultured on SIM for 0 day, 4 days, 6 days, 10 days and 14 days. Scale bars, 1 mm.

Comparable maximal percentages of shoot primordia were reached after 18 days of incubation on SIM for both wild type and all tested mutants, including *met1*, *kyp*, *jmj14* and *hac1* (Figure 1A–1C), indicating that there was no significant difference in the capacities of *de novo* shoot regeneration. However, it took different induction time for the wild-type calli and the mutant calli to reach half of the maxima (Figure 1A–1C). Specifically, the mutants whose epigenetic changes were associated with more active transcription, such as *met1*, *kyp*, *jmj14* [27]–[29], took significantly less time to reach half of the maxima as compared to the wild type (Figure 1A–1C). In contrast, the mutant associated with more repressed transcription such as *hac1* took significantly more time to reach half of the maxima (Figure 1C). We obtained similar results indicating precocious or delayed initiation of shoots in these mutants using either pistils or roots as explants (Figure 1A–1C, Figure S1). Interestingly, calli of *met1* cultured on SIM develop differently from those of the wild type (Figure 1D). At 4 days on SIM, around 70% *met1* calli contained green regions from which the shoots would differentiate, but these green regions could not be identified in the wild-type calli. At 6 to 14 days on SIM, more shoots emerged from the *met1* calli than those from the wild-type calli (Figure 1D). At 18 days on SIM, the shoots from the *met1* calli were much precocious compared with those from the wild-type calli although the percentages of shoots from both the wild-type and the *met1* calli were similar (Figure 1A). We also obtained similar results with roots as explants (Figure S2). Thus, these results indicated that epigenetic modifications, including DNA methylation and histone modifications, played roles in mediating developmental rate of *de novo* shoot regeneration.

### Regulation of *WUS* expression during *de novo* shoot regeneration may have resulted from dynamic DNA methylation

**Figure 2:**
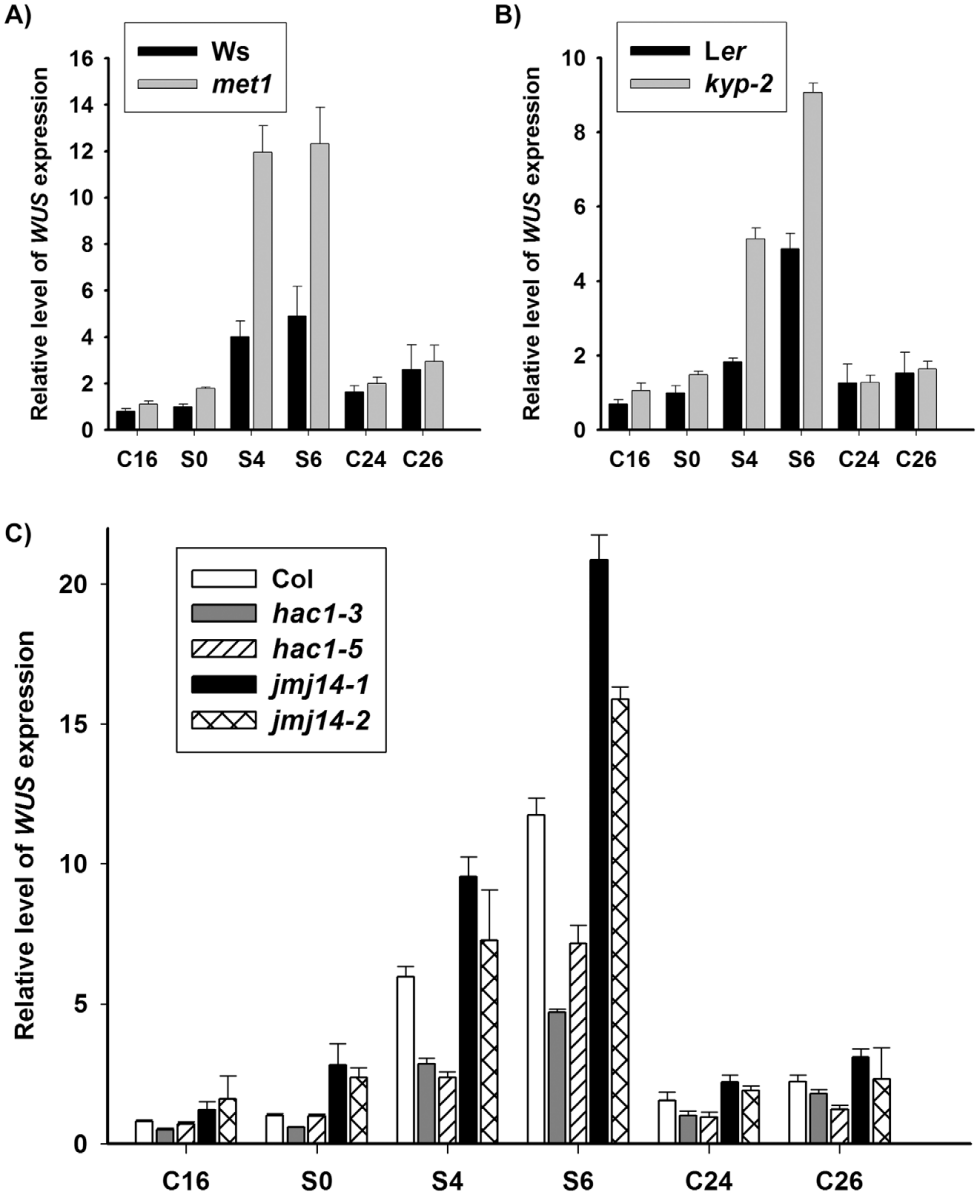
DNA methylation and histone modifications regulate *WUS* transcript levels. A) Transcript levels of *WUS* in calli of the wild type (Ws) and the mutant *met1*. B) Transcript levels of *WUS* in calli of the wild type (L*er*) and the mutant *kyp-2*. C) Transcript levels of *WUS* in calli of the wild type (Col) and the mutants, *hac1-3*, *hac1-*5, *jmj14-1* and *jmj14-2*. Total RNAs were isolated from calli of wild type (Ws, L*er* and Col) and various mutants (*met1*, *kyp-2*, *jmj14-1*, *jmj14-2*, *hac1-3* and *hac1-5*) cultured on SIM at the indicated time points, respectively. *WUS* transcript levels were quantified by qRT-PCR. The results are shown as mean values of three biological replicates with standard errors. The relative expression level of *WUS* gene, corresponding to the expression level of *TUBULIN2*, was calculated using the comparative C(T) method. After incubating on CIM for 20 days (S0), some of the calli were transferred onto SIM for further induction for 4 days (S4) and 6 days (S6), other calli were still cultured on CIM as controls (C24, C26). C16, C24, C26 indicated that pistils as explants were cultured on CIM for 16 days, 24 days and 26 days, respectively.

**Figure 3:**
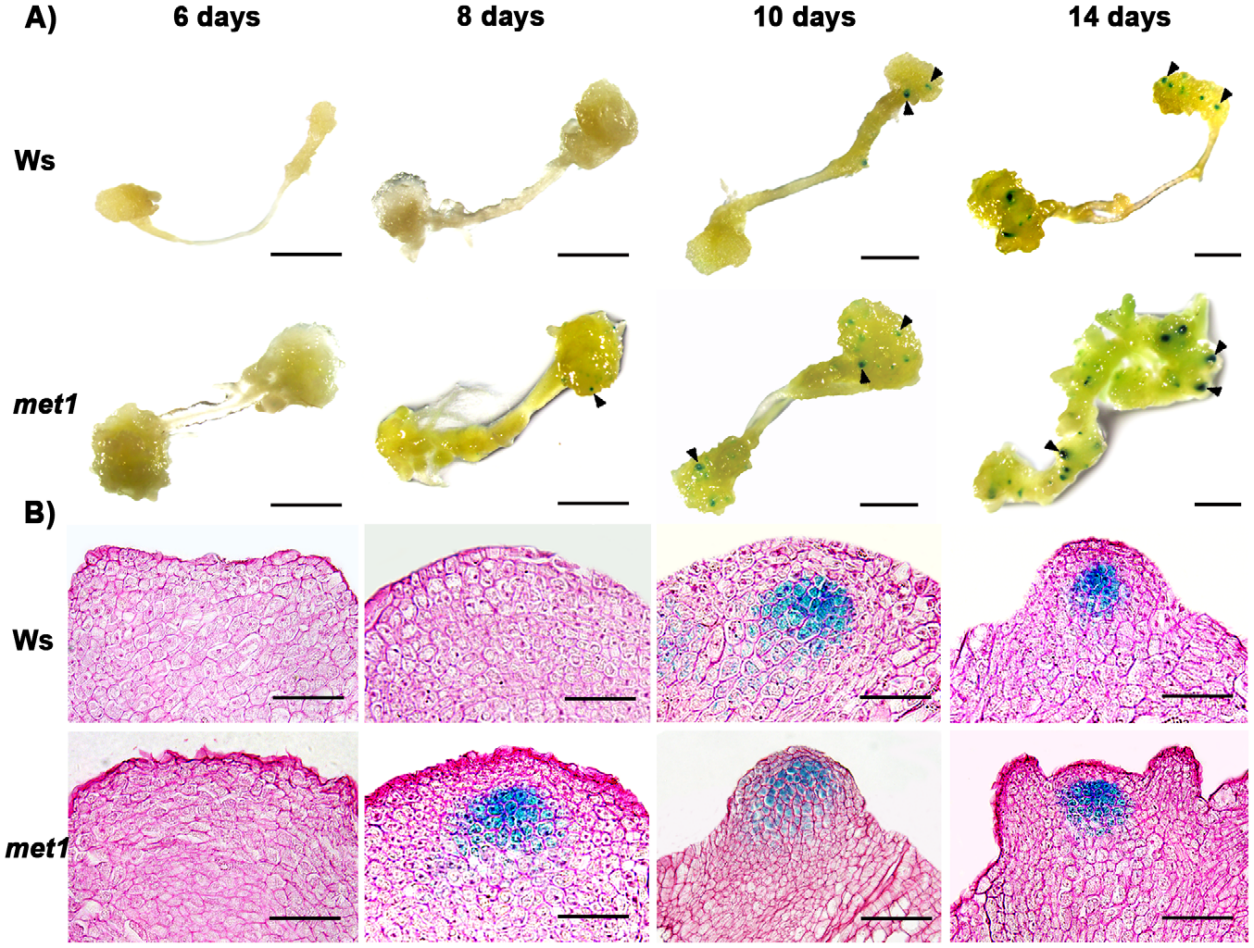
Regulation of *WUS* expression in *met1* mutant. A) By roots as explants, *pWUS::GUS* transgenic calli in the wild type transferred onto SIM for 6 days, 8 days, 10 days and 14 days, and *pWUS::GUS* transgenic calli in the *met1* mutant transferred onto SIM for 6 days, 8 days, 10 days and 14 days. Arrowheads indicate *pWUS::GUS* signals. Scale bars, 1 mm. B) Longitudinal sections of *pWUS::GUS* transgenic calli in both the wild type and the *met1* mutant transferred onto SIM for 6 days, 8 days, 10 days and 14 days, respectively. Scale bars, 50 µm.

It was well established that *WUS* expression is critical for stem cell formation during *de novo* shoot regeneration [5], [6]. Here, we showed that induction of wild-type calli on SIM for 4 days (S4) and 6 days (S6) was accompanied by a significant increase of *WUS* level through qRT-PCR analysis (Figure 2A). In contrast, *WUS* transcripts were in a low level in wild-type calli on CIM for 16 days (C16) and 20 days (S0, non-induced calli), and similar results were obtained in the prolonged time, such as calli on CIM for 24 days (C24) and 26 days (C26). We further determined the expression patterns of *WUS* by *pWUS::GUS* reporter and *in situ* hybridization, and the results demonstrated that local distribution of *WUS* transcripts occurred in wild-type calli on SIM (Figure 3, Figure S3). Because it was shown previously that *WUS* expression was mediated by epigenetic factors [10], we were tempted to hypothesize that the regulation of *WUS* expression during *de novo* shoot regeneration might have resulted from reduced DNA methylation.

**Figure 4:**
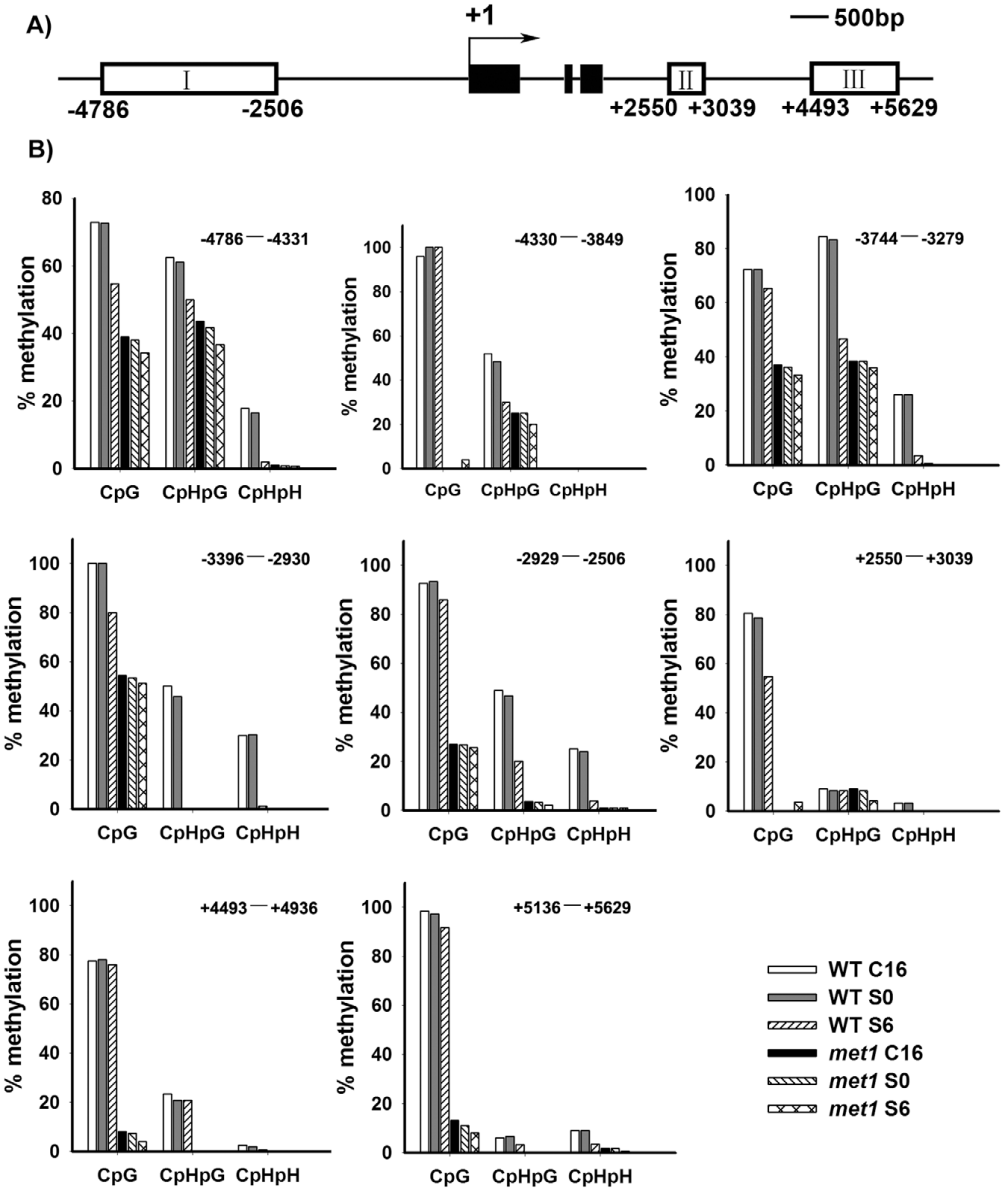
Analysis of *WUS* methylation through bisulfite genomic sequencing. A) A diagram of *WUS* structure, with +1 as the transcription start site and rectangles representing the methylated region I, II and III. B) Cytosine methylation at region I, II and III of *WUS* was determined by bisulfite genomic sequencing. Genomic DNA methylation status of *WUS* is shown in calli of the wild type on CIM for 16 days (WT, C16) and for 20 days (WT, S0), and on SIM for 6 days (WT, S6). Calli of *met1* are incubated on CIM for 16 days (*met1*, C16) and for 20 days (*met1*, S0), and on SIM for 6 days (*met1*, S6). H represents A, T or C.

To test this possibility, we first compared DNA methylation of the ∼10 kb *WUS* genomic sequences between the calli of wild type on CIM (C16 and S0) and those on SIM (S6) by bisulfite genomic sequencing. Three regions within the *WUS* genomic sequences were hyper-methylated in S0 calli but substantially decreased in S6 calli (Figure 4A and 4B). Among the three regions, region I was previously proposed to regulate *WUS* expression [36]. Both CpG dinucleotide motifs and non-CG motifs in the three regions of the *WUS* genomic sequences showed induced demethylation upon induction on SIM (Figure 4B). These results showed that *de novo* shoot regeneration was accompanied with demethylation on methylated *WUS* genomic sequences. That could partially contribute to the regulation of *WUS* expression during *de novo* shoot regeneration.

### Demethylation and regulation of *WUS* expression in *met1* mutant

Because DNA methylation was significantly reduced in *met1* mutant [27], we wondered whether DNA methylation in the *WUS* genomic sequences would be affected in *met1* mutant. To find out, we used two approaches. First, we compared the expression patterns of *WUS* in wild-type calli and *met1* calli at different induction points. Indeed, the *met1* mutant showed much higher *WUS* level than that in the wild type at each time point by qRT-PCR (Figure 2A). Then, *in situ* hybridization analysis demonstrated that localization of *WUS* in the *met1* calli on SIM was earlier than that in the wild-type calli on SIM (Figure S3A–S3F, Table S1). *GUS* staining confirmed that the pattern of *WUS* expression is similar to that *in situ* hybridization (Figure 3), and the number of *GUS* signal distribution in both the *met1* calli and the wild-type calli on SIM is consistent to percentages of shoot primordia on SIM at different induction points (Figure 3, Figure S3, Table S2). Thus, the results indicated that *WUS* expression and corresponding developmental rate of *de novo* shoot regeneration were mediated by reduced DNA methylation.

Next, we tested whether *MET1* loss of function affected the methylation status of *WUS* genomic region by bisulfite genomic sequencing. We found that the calli of *met1* mutant on CIM (C16 and S0) and on SIM (S6) showed much lower level of DNA methylation in the *WUS* genomic region than those of wild type under the same condition (Figure 4B). *WUS* expression was detected in *met1* calli earlier than in wild type based on *in situ* hybridization and GUS reporter analysis (Figure 3 and Figure S3). In addition, *met1* contained more *WUS*-expressing regions than wild type, indicating that increased *WUS* expression level contributed to elevated the number of organizing centers (Figure 3 and Figure S3). These results suggested that the regulation of *WUS* expression in *met1* mutant during *de novo* shoot regeneration could at least partially be contributed by DNA demethylation on methylated *WUS* genomic sequences.

### Dynamic changes of histone modifications at the genomic regions of *WUS* during *de novo* shoot regeneration

Higher *WUS* level in the *met1* mutant suggested the involvement of *MET1*-mediated DNA methylation in the regulation of *WUS* expression. However, the expression of *WUS* still responded to the induction by incubation on SIM in *met1* mutant (Figure 2A), indicating additional pathways that regulated the dynamic expression of *WUS*. Because we showed that histone modifications were also important for *de novo* shoot regeneration (Figure 2B and 2C), we next tested whether histone modifications played a role in mediating *WUS* expression during *de novo* shoot regeneration.

We analyzed several histone modifications for the *WUS* genomic sequences using chromatin immunoprecipitation at two developmental stages: S0 and S6. Methylation at histone H3 at lysine 4 (H3K4me3) was shown to occur in euchromatin undergoing active transcription [37]. Whereas methylation at histone H3 at lysine 9 (H3K9me2) was shown to inhibit transcription [38]. Additionally, acetylation at histone H3 at lysine 9 (H3K9ac) is one of the most characterized epigenetic marks invariably associated with active transcription in all species investigated so far [18]. It also plays a crucial role in plant development [39].

**Figure 5:**
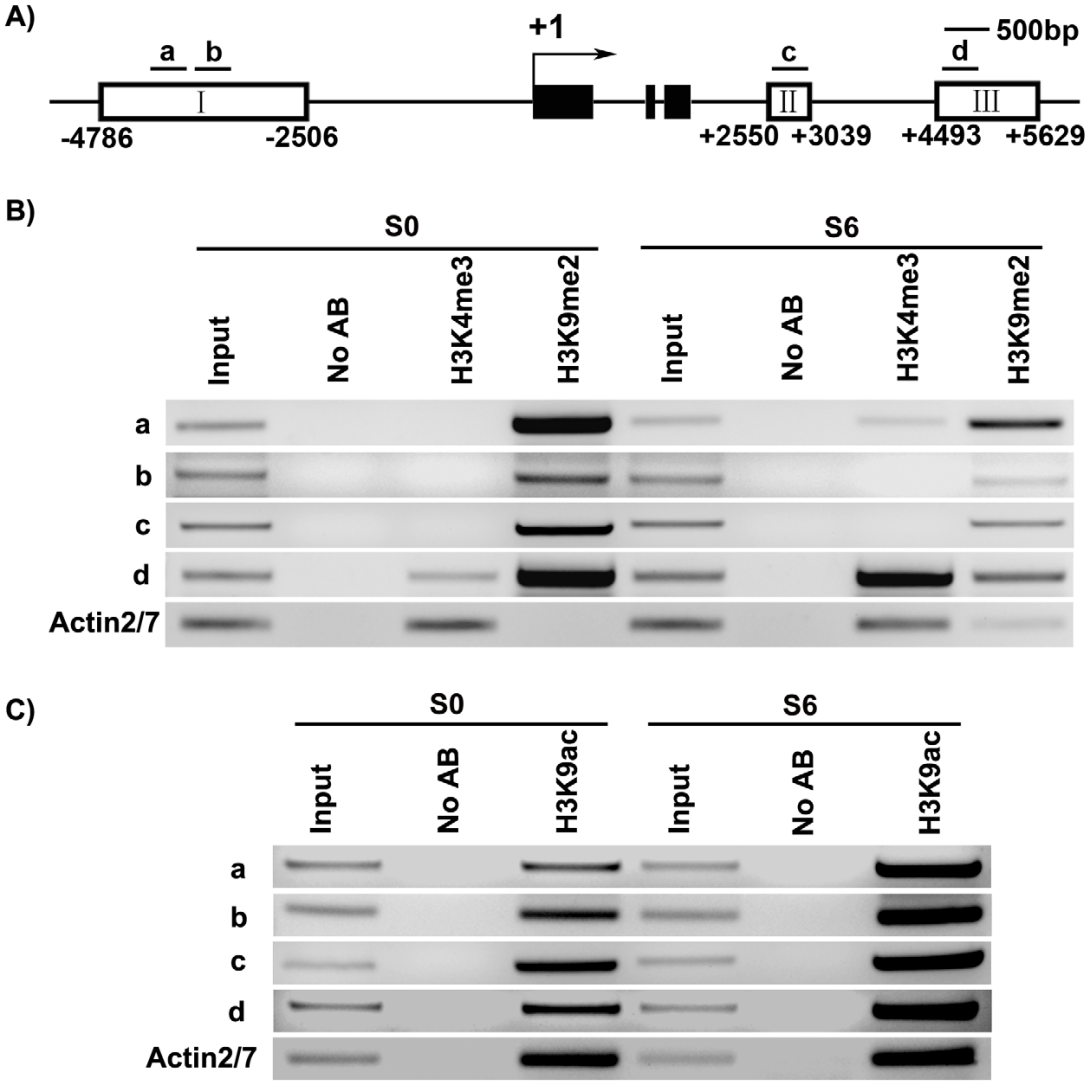
ChIP assays of calli of wild type on SIM at the *WUS* locus. A) A diagram of *WUS* structure, with +1 as the transcription start site, and bars representing the regions examined by ChIP. B) ChIP analysis using antibodies against trimethyl H3K4 and dimethyl H3K9 at 5′ and 3′ regions of *WUS* in calli of wild type for 20 days on CIM (S0) and 6 days on SIM (S6). C) ChIP analysis using antibodies against H3 acetyl Lys 9 at 5′ and 3′ regions of *WUS* in calli of wild type (S0, S6). *ACTIN* was used as a control. The input was chromatin before immunoprecipitation. ‘No AB’ corresponds to chromatin treated with normal mouse IgG as the negative control. Three biological replicates were analyzed and each was tested by three technical replicates, and similar results were obtained. Representative data were shown.

Our results showed that these three histone modifications were dynamically regulated at the *WUS* genomic sequences during *de novo* shoot regeneration. Compared with S0, S6 showed an increase in the levels of H3K4me3 at region a and d, but not at b and c (Figure 5A and 5B). H3K4me3 occurred in euchromatin undergoing active transcription [37], therefore increased H3K4me3 levels were consistent with *WUS* induction during *de novo* shoot regeneration (Figure 1C, Figure 2C). A mark for chromatin acetylation, H3K9ac, also showed increased levels at all four regions during induction (Figure 5C). In contrast to these epigenetic marks associated with active transcription, H3K9me2, which is associated with transcription suppression [37] were reduced during *de novo* shoot regeneration in all four regions (Figure 5B). The changes at these epigenetic marks around *WUS* genomic region explained the active state of *WUS* chromatin structure, and might well contribute to the regulation of *WUS* expression during *de novo* shoot regeneration.

### *WUS* expression was changed in mutants defective in histone modifications

Dynamic histone modifications at the genomic regions of *WUS* during *de novo* shoot regeneration indicated that histone modifications contributed to regulation of *WUS* expression during *de novo* shoot regeneration. To provide further evidence that histone modifications regulated *WUS* expression in this process, we examined transcript level of *WUS* in mutants that were defective in histone modifications by qRT-PCR. As stated before, *KYP*, *JMJ14* and *HAC1* encoded enzymes for histone modification, mutations of which affected the developmental rate of *de novo* shoot regeneration (Figure 1B and 1C, Figure S1). Comparing with the wild-type calli, levels of *WUS* expression in the calli of the mutant *kyp-2* were significantly enhanced compared to those of wild type for 6 days on SIM (Figure 2B). Similar results were obtained for the mutants *jmj14-1* and *jmj14-2* (Figure 2C). Contrast to the mutants *kyp* and *jmj14*, the levels of *WUS* transcripts in two different allelic *hac1* mutants were reduced compared to that of wild type (Figure 2C).

Then, we used *kyp-2* calli on SIM (S0, S4, and S6) to do *in situ* hybridization analysis. The results showed that localization of *WUS* signals in *kyp-2* calli on SIM occurred early comparing to that in wild-type calli on SIM (Figure S3G–S3L). Also, the number of localized *WUS* signals in *kyp-2* calli on SIM (S4 and S6) was more than that in wild-type calli at the same time points (Table S1). Similar to the case of *met1*, expression of *WUS* appeared earlier in *kyp-2* calli than in wild type (Figure S3). Thus, changes of *WUS* expression in these mutants correlated with their different developmental rates of *de novo* shoot regeneration, suggesting that *WUS* expression was regulated by histone modifications.

### SIM-induced as well as *MET1*-dependent transcriptional changes during *de novo* shoot regeneration

**Figure 6:**
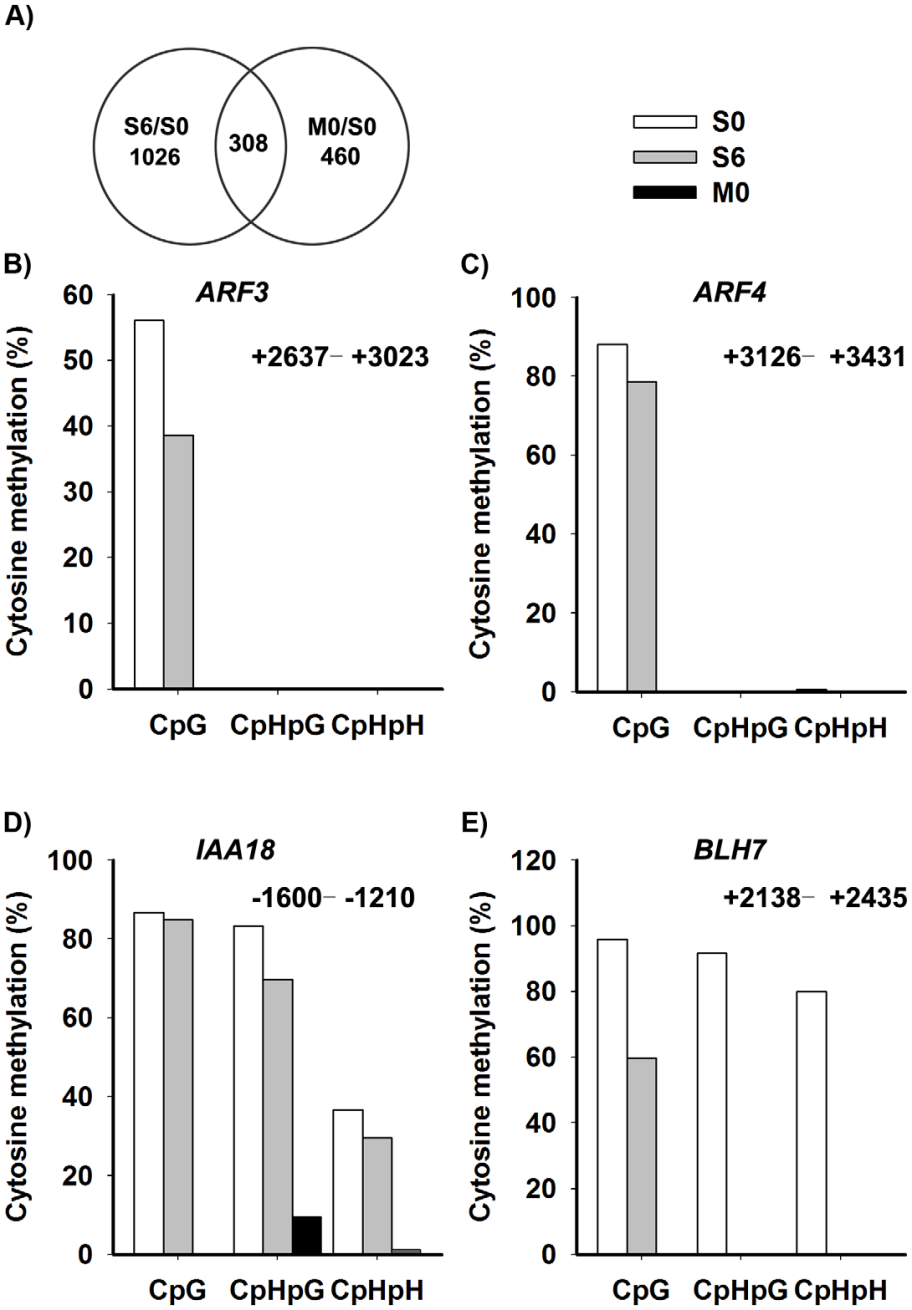
Identification of the candidate genes regulated by DNA methylation. A) The overlap between differentially-expressed genes of S6 versus S0 (Table S3) and M0 versus S0 (Table S4) were identified as candidate genes, and were listed in Table S5. A two-fold difference in the expression level of genes with a *q* value≤0.05 between S6 versus S0 and M0 versus S0 was set as the threshold for the selection of differentially-expressed genes. B)–E) Cytosine methylation levels of *ARF3*, *ARF4*, *IAA18* and *BLH7* genes in calli of wild type (S0, S6), and calli of *met1* (M0) were determined by bisulfite genomic sequencing. H represents A, T or C.

Our results showed that DNA methylation and histone modifications regulated *WUS* expression during *de novo* shoot regeneration. To get a whole picture of epigenetic modifications during this process, we decided to do a genome-wide expression profiling using the Affymetrix ATH1 full genome array. We analyzed the transcriptomes of wild-type calli being transferred to CIM for 20 days (S0) and to SIM for 6 days (S6). Because *met1* calli showed significantly different developmental rate from wild-type calli, we also analyzed transcriptomes of *met1* calli being transferred to CIM for 20 days (M0) for comparison. Significance Analysis of Microarrays software package analysis was conducted for three biological samples replicates between the Ws and *met1*. The *q* value≤0.05 and fold change ≥2 were used as the threshold for candidate gene selection (Figure 6A). This criterion gave 1334 upregulated genes, and 501 downregulated genes by induction on SIM (S6 versus S0) (Table S3). 768 candidate genes showed over 2 fold difference between M0 and S0, suggesting that they might be regulated by MET1-dependent DNA methylation (Table S4). 308 candidate genes showed over 2 fold difference both between S6 versus S0 and between M0 versus S0, suggesting that they might be induced on SIM and be regulated by MET1-dependent DNA methylation (Table S5). By qRT-PCR analysis, we confirmed the microarray data (Figure S4).

Because auxin and cytokinin are essential for *de novo* shoot regeneration [2], [5], we selected genes involved in cytokinin and auxin signaling for bisulfite sequencing analysis. Indeed, some displayed differential methylation patterns during *de novo* shoot regeneration, such as *AUXIN RESPONSE FACTOR3* (*ARF3*), *AUXIN RESPONSE FACTOR4* (*ARF4*), *INDOLE-3-ACETIC ACID INDUCIBLE18* (*IAA18*) and *BELL1-LIKE HOMEODOMAIN7* (*BLH7*) (Figure 6B–6E). A loss of DNA methylation occurred in these genes, along with increased levels of their transcription in induced wild-type calli (Figure S4). Their expression levels were also higher in *met1* than those in the wild type, suggesting that the expression of these genes might be regulated by a MET1-dependent dynamic DNA methylation during shoot regeneration. On the other hand, some candidate genes selected from SIM-induced and MET1-dependent pathways displayed no methylation, such as *ASMMETRIC LEAVES1* (*AS1*), *ARABIDOPSIS RESPONSE REGULATOR15* (*ARR15*), *CYTOKININ OXIDASE/DEHYDROGENASE1* (*CKX1*), *INDOLE-3-ACETIC ACID27* (*IAA27*) and *PINOID2* (*PID2*), but they displayed great changes in their transcriptional levels upon SIM-induction, implying that those genes might not be directly regulated by MET1 (Table S5).

### Epigenetic modifications: evolutionary recurring themes for reprogramming

DNA methylation and histone modifications are critical epigenetic processes that control chromatin structure and gene expression during development and differentiation [17], [18], and there are likely complicated interactions between these processes [20], [40]. In human, a crosstalk between DNA methylation and histone modifications has been proposed to regulate gene transcription in tumors [20]. Similarly, DNA methylation controls histone H3K9 methylation and further affect heterochromatin assembly in *Arabidopsis* [41]. Recent study has indicated that chromatin status facilitates the accessibility of transcription factor to *FLOWERING LOCUS T* (*FT*) in *Arabidopsis*, and distant regulatory regions are required for *FT* transcription [42]. *WUS* transcription is regulated through a fairly complicated chromatin remodeling mechanism in the SAM of the *Arabidopsis* plant [43]. It was shown that *WUS* expression was positively correlated with FASCIATA1 (FAS1)/FAS2, subunits of ASSEMBLY FACTOR-1 (CAF-1), and BRUSHY1 (BRU1), both of which regulate post-replicative stabilization of chromatin structure [44], [45]. Another study showed that the chromatin remodeling factor SPLAYED (SYD) directly regulated *WUS* to maintain proper *WUS* transcript levels in its spatial expression domain [46]. It has been demonstrated that at least 3.5 kb fragment upstream of *WUS* is required for its spatiotemporal expression during plant development [36]. Here, we showed that the 5′ and 3′ regions of *WUS* were regulated by SIM-induced changes of DNA methylation and histone modifications. Because the *met1-3 kyp-7* double mutant displayed more severe phenotypes than each single mutant [19], we propose that regulation of *WUS* by DNA methylation and histone modifications may function in a partially redundant manner during *de novo* shoot regeneration. To understand mechanism of the *in vitro* organogeneis mediated by the factors involved in both DNA methylation and histone modifications, knocking out both DNA methylation and histone modifications remains to be investigated in the future.

It has long been thought that animal cells, once committed to a specific lineage, can no longer change their fate. However, recent studies suggested that differentiated animal cells do maintain plasticity and can be induced to undergo reprogramming [47], [48]. Further studies have shown that differentiated cells in mouse can be reprogrammed to pluripotent stem cells by introducing four transcription factors [49]. Plant cells can easily regenerate organs from the differentiated tissues under proper cultured conditions [1]. Previously, we used *Arabidopsis* ptstils as explants on CIM to obtain the callus, a mass of pluripotent cells [26], and by transferring calli onto SIM, the expression of *WUS* was induced in a group of cells termed the organizing center as a self-renewing source of stem cells within calli. The induced organizing center and stem cells were responsible for subsequent shoot regeneration. Here, we showed that expression of many genes was induced by SIM-induction (Figure 6A). Those genes were divided into either MET1-dependent or MET1-independent. Among MET1-dependent genes, WUS is a key transcription factor to regulate shoot regeneration [1]. ARF3 was required for shoot induction (Cheng et al., unpublished data). Previous study showed that ARF3 and ARF4 act redundantly to establish the abaxial cell fate of the *Arabidopsis* leaves [50]. Thus, ARF3 and ARF4 may function on *de novo* meristem formation mediated by epigenetic modifications. MET1-independent genes might also be involved in the process of shoot induction. Our results suggested that pluripotent cells of the callus can be reprogrammed to stem cells and subsequent, shoot formation through the regulation of both MET1-dependent genes, such as *WUS* and *ARFs*, and some MET1-independent genes.

In conclusion, our results indicate that dynamic DNA methylation and histone modifications contribute to the control of stem-cell formation and subsequent shoot regeneration. These epigenetic modifications regulate *WUS* and probably hormone-related genes, whose spatiotemporal expression was critical for *de novo* shoot regeneration. In mammals, epigenetic modifications of transcription factors and of components in hormone signaling pathways also play crucial roles in cell differentiation and organogenesis [51], [52]. Our results thus provide an interesting scenario in which epigenetic modifications were adopted as recurring themes during evolution for *de novo* organogenesis.

## Materials and Methods

### Plant materials

The *met1* mutant in the Wassilewskija (Ws) background was a kind gift from Dr. J. Bender (The MCB Department of Brown University) [27]. The *kyp-2* [28] mutant in the Landsberg (L*er*) background, *jmj14-1*, *jmj14-2* [29], *hac1-3*, and *hac1-5* [31] mutants in the Columbia (Col) background were generously provided by Dr. Xiaofeng Cao (Institute of Genetics and Developmental Biology, Chinese Academy of Sciences).

### Plant growth and shoot regeneration

Plants were grown as previously described [9]. *Arabidopsis* seeds were surface sterilized and plated on germination medium [53]. After cold treatment for 2 days at 4°C in the dark, they were transferred to sterile conditions or the growth chamber at 22°C in a 16 h light/8 h dark cycle. Shoot regeneration procedures used in this study were based on the previously described protocols [26], [54]. Pistils were excised from sterile *Arabidopsis* plants and transferred onto callus induction medium (CIM, MS medium [53] with 0.5 mg/L 2, 4-dichlorophenoxyacetic acid (2, 4-D) and 1.0 mg/L 6-benzylaminopurine (6-BA)). The explants were incubated for 20 days on CIM to induce callus production, and calli were then transferred onto shoot induction medium (SIM, MS medium with 0.01 mg/L indole-3-acetic acid (IAA) and 2 mg/L zeatin (ZT)). Root explants of 5–10 mm length were excised from 7-day-sterile seedlings, then transferred onto callus induction medium (CIM, Gamborg's B5 medium [55] with 0.5 g/L MES, 2% glucose, 0.2 µmol/L kinetin, and 2.2 µmol/L 2,4-dichlorophenoxyacetic acid (2,4-D), 0.8% agar), and incubated for 6 days in continuous light. Finally, explants were transferred onto shoot-inducing medium (SIM, Gamborg's B5 medium with 0.5 g/L MES, 2% glucose, 0.9 µmol/L 3-indoleacetic acid, 0.5 µmol/L 2-isopentenyladenine) and incubated in continuous light.

The morphology of calli was examined and photographed with an Olympus microscope. We defined the number of regenerated shoots as the number of at least 2 mm long shoots on each callus.

### *In situ* hybridization

Probes were labeled using digoxigenin RNA labeling kit (Boehringer Mannheim). An antisense probe from a full-length *WUS* cDNA clone was generated using T7 RNA polymerase, and a sense probe was synthesized using SP6 RNA polymerase. The detailed protocol was carried out as described previously [56]. Primer sequences used for probes amplification are summarized in Table S6.

### β-glucuronidase (GUS) assay

Plant tissues were incubated in GUS assay solution (50 mmol/L Na_2_HPO_4_, 50 mmol/L KH_2_PO_4_, pH 7.2, 10 mmol/L Na_2_EDTA, 0.5 mmol/L K_3_Fe(CN)_6_, 0.5 mmol/L K_4_Fe(CN)_6_, 1% Triton X-100 and 2 mmol/L X-Gluc (Bio. Basic Inc., Canada)) at 37°C for 12 h. To further investigate *WUS* expression pattern, some GUS-stained tissues were embedded in paraffin (Sigma) and sectioned. To display the outline of cells clearly, ruthenium red (200 mg/L) was used to stain cell walls.

### Genomic bisulfite sequencing

DNA methylation assays were performed by bisulfite sequencing as previously described [57]. PCR products were cloned into the pMD19-T Simple Vector (Takara), and 12 clones were sequenced to determine the methylation status of a locus in each genotype. Primer sequences are shown in Table S6. Bisulfite sequencing data were analyzed by the CyMATE software [58]. The results returned by CyMATE were input into SigmaPlot 10.0 to illustrate DNA methylation frequencies at CG, CHG and CHH (where H = A, C or T) at the various cultured stages of each genotype.

### Chromatin immunoprecipitation assay

The *Arabidopsis* calli grown on CIM for 20 days (S0) and on SIM for 6 days (S6) were vacuum-infiltrated with formaldehyde crosslinking solution. Chromatin immunoprecipitation was performed according to manufactures' instructions (Epigentek Group Inc. USA, Catalogno. P-2014). Chromatin samples were immunoprecipitated with antibodies against a negative control normal mouse IgG and H3 dimethyl Lys 9 (both included in EpiQuik™ Plant ChIP Kit), or with antibodies against H3 trimethyl Lys 4 (Abcam USA, Catalogno. ab1012) and H3 acetyl Lys 9 (Abcam USA, Catalogno. ab10812). PCR amplification was performed in 25 µL volumes for 32 to 37 cycles to determine the appropriate conditions for the PCR products of each region. Primer sequences are shown in Table S6. The PCR products were electrophoresed in a 2% agarose gel. Three biological replicates were analyzed and each was tested by three technical replicates.

### Total RNA isolation and quantitative real-time PCR analysis

Total RNAs were isolated from callus tissues 2 to 3 mm deep from the surface. Quantitative real-time PCRs (qRT-PCRs) were performed as described previously [9]. To check the specificity of amplification, the melting curve of the PCR products was detected. The expression levels of specific genes were standardized to the housekeeping gene *TUBULIN2*. Each reaction was carried out in three biological replicates. The relative expression level of each gene, corresponding to the expression level of *TUBULIN2*, was calculated using the comparative C_T_ method [59]. Primer sequences used for qRT-PCR are summarized in Table S6.

### DNA microarray analysis

RNA of three plant samples was prepared from each of the following tissue types: the wild-type calli cultured on CIM for 20 days (S0), and on SIM for 6 days (S6); the *met1* mutant calli cultured on CIM for 20 days (M0). RNA purification, probe labeling, chip hybridization, probe array scanning and data pre-processing normalization were performed by the Affymetrix custom service (CapitalBio, Beijing, China). Significance Analysis of Microarrays software package analysis was conducted for three biological samples replicates between the Ws and *met1*. When all replicates clustered together, further analysis was performed based on mean values. A two-fold change in the gene expression levels between one versus another samples with a *q* value≤0.05 was set as the threshold for altered gene expression. Microarray data are available in the ArrayExpress database (www.ebi.ac.uk/arrayexpress) under accession number E-MEXP-3120.

## Supporting Information

Figure S1 Frequency of shoot regeneration of *met1* mutant and the mutants defective in histone modifications. Frequency of shoot regeneration of the wild type (Col) and the mutants *jmj14-2* and *hac1-5* was shown, using pistils as explants. Frequency of shoot regeneration of the wild type (Ws, L*er* and Col) and the mutants *met1*, *kyp-2*, *jmj14-1*, *jmj14-2*, *hac1-3* and *hac1-5* was shown, using roots as explants. Standard errors were calculated from three sets of biological replicates. In each replicate, at least 60 calli were examined.
(TIF)

Figure S2 *MET1* mutation promotes shoot regeneration in *Arabidopsis* using roots as explants. Calli of the wild type (Ws) and the *met1* mutant were cultured on SIM for 6 to 18 days. Scale bars, 1 mm.
(TIF)

Figure S3 Expression patterns of *WUS* were changed in *met1* and *kyp-2* mutants. *In situ* hybridization of *WUS* expression in calli of the wild type (Ws) cultured on SIM for A) 0 day, B) 4 days and C) 6 days, and that of *met1* mutant cultured on SIM for D) 0 day, E) 4 days and F) 6 days. *In situ* hybridization of *WUS* expression in calli of the wild type (L*er*) cultured on SIM for G) 0 day, H) 4 days and I) 6 days, and that of *kyp-2* mutant cultured on SIM for J) 0 day, K) 4 days and L) 6 days. Scale bars, 50 µm.
(TIF)

Figure S4 Expression patterns of candidate genes validated by qRT-PCR. Total RNAs were isolated from calli of wild type and *met1* cultured on SIM at the indicated time points, and the transcripts of genes *ARF3*, *ARF4*, *IAA18*, *BLH7*, *ANT*, *AS1*, *CKX1*, and *ARR15* were measured by qRT-PCR. Three independent RNA preparations were analyzed for each time point. Mean values were calculated from triplicate qRT-PCR analysis with standard errors. The relative expression level of each gene, corresponding to the expression level of *TUBULIN2*, was calculated using the comparative C(T) method.
(TIF)

Table S1 The percentage of the calli with *WUS* expressing signals detected by *in situ* hybridization.
(DOC)

Table S2 The number of *pWUS::GUS* signal distribution detected in each callus.
(DOC)

Table S3 List of 1334 up-regulated genes and 501 down-regulated genes in S6 as compared to S0.
(XLS)

Table S4 List of 768 genes showing more than two-fold difference between M0 and S0.
(XLS)

Table S5 List of 308 genes showing more than two fold difference both between S6 and S0 and between M0 and S0.
(XLS)

Table S6 Sequences of primers used in this study.
(XLS)
