# Supplementary figures and images for: DNA Methylation and Histone Modifications Regulate De Novo Shoot Regeneration in Arabidopsis by Modulating WUSCHEL Expression and Auxin Signaling
Source: PLoS Genet. 2011 Aug 18;7(8):e1002243. doi: 10.1371/journal.pgen.1002243 (PMC3158056; doi:10.1371/journal.pgen.1002243)

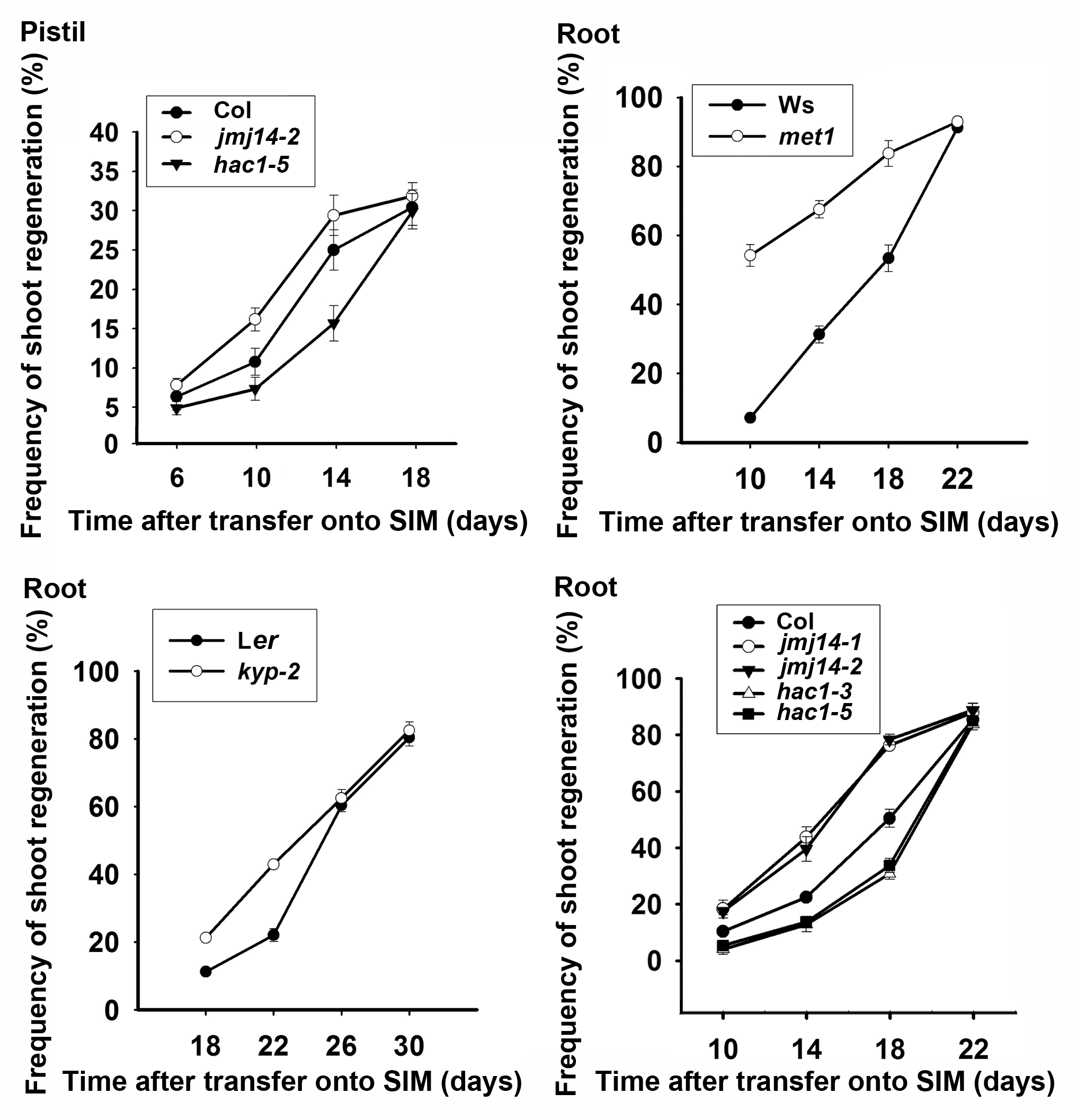

Supplement: Figure S1 — Frequency of shoot regeneration of met1 mutant and the mutants defective in histone modifications. Frequency of shoot regeneration of the wild type (Col) and the mutants jmj14-2 and hac1-5 was shown, using pistils as explants. Frequency of shoot regeneration of the wild type (Ws, Ler and Col) and the mutants met1, kyp-2, jmj14-1, jmj14-2, hac1-3 and hac1-5 was shown, using roots as explants. Standard errors were calculated from three sets of biological replicates. In each replicate, at least 60 calli were examined. (TIF) [file pgen.1002243.s001.tif]

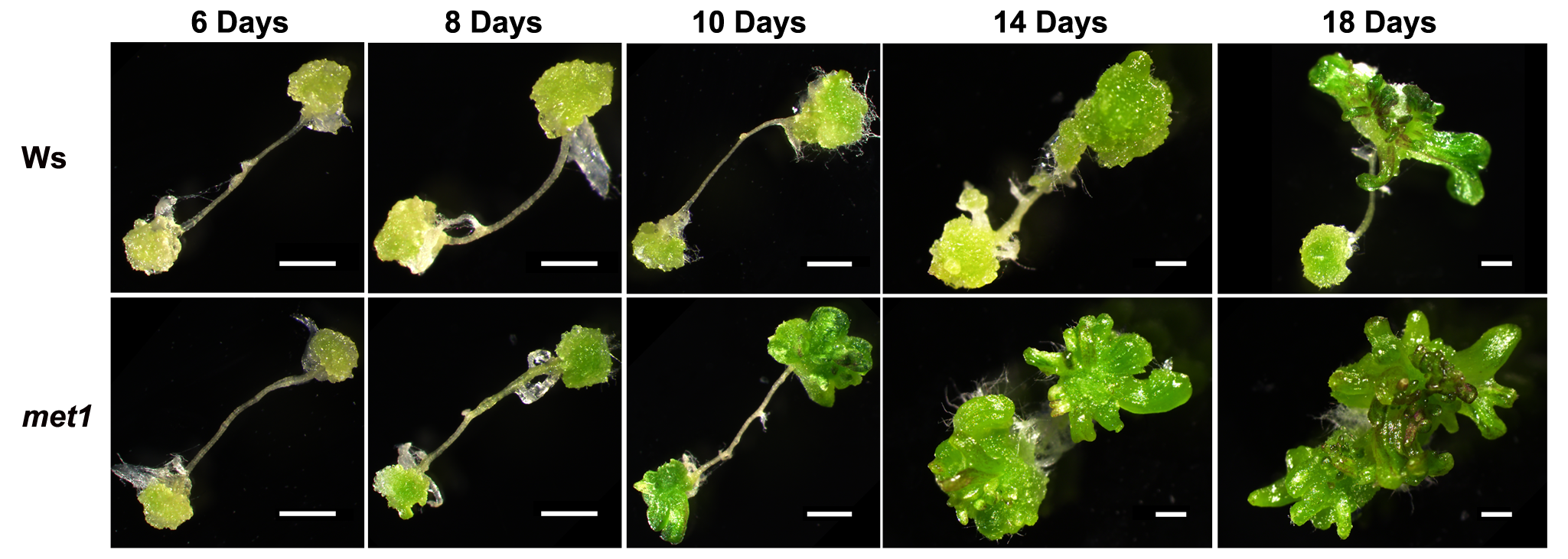

Supplement: Figure S2 — MET1 mutation promotes shoot regeneration in Arabidopsis using roots as explants. Calli of the wild type (Ws) and the met1 mutant were cultured on SIM for 6 to 18 days. Scale bars, 1 mm. (TIF) [file pgen.1002243.s002.tif]

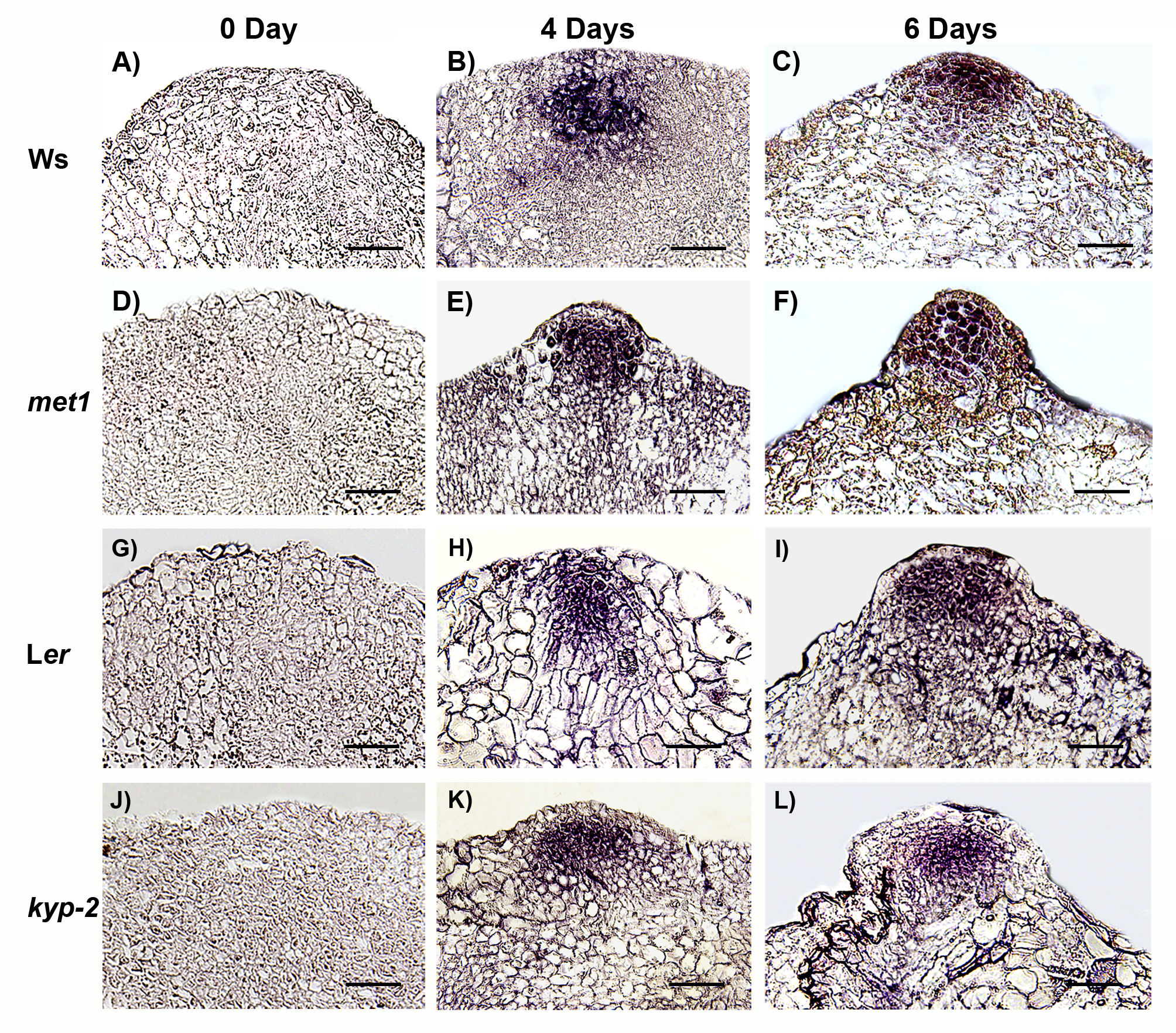

Supplement: Figure S3 — Expression patterns of WUS were changed in met1 and kyp-2 mutants. In situ hybridization of WUS expression in calli of the wild type (Ws) cultured on SIM for A) 0 day, B) 4 days and C) 6 days, and that of met1 mutant cultured on SIM for D) 0 day, E) 4 days and F) 6 days. In situ hybridization of WUS expression in calli of the wild type (Ler) cultured on SIM for G) 0 day, H) 4 days and I) 6 days, and that of kyp-2 mutant cultured on SIM for J) 0 day, K) 4 days and L) 6 days. Scale bars, 50 µm. (TIF) [file pgen.1002243.s003.tif]

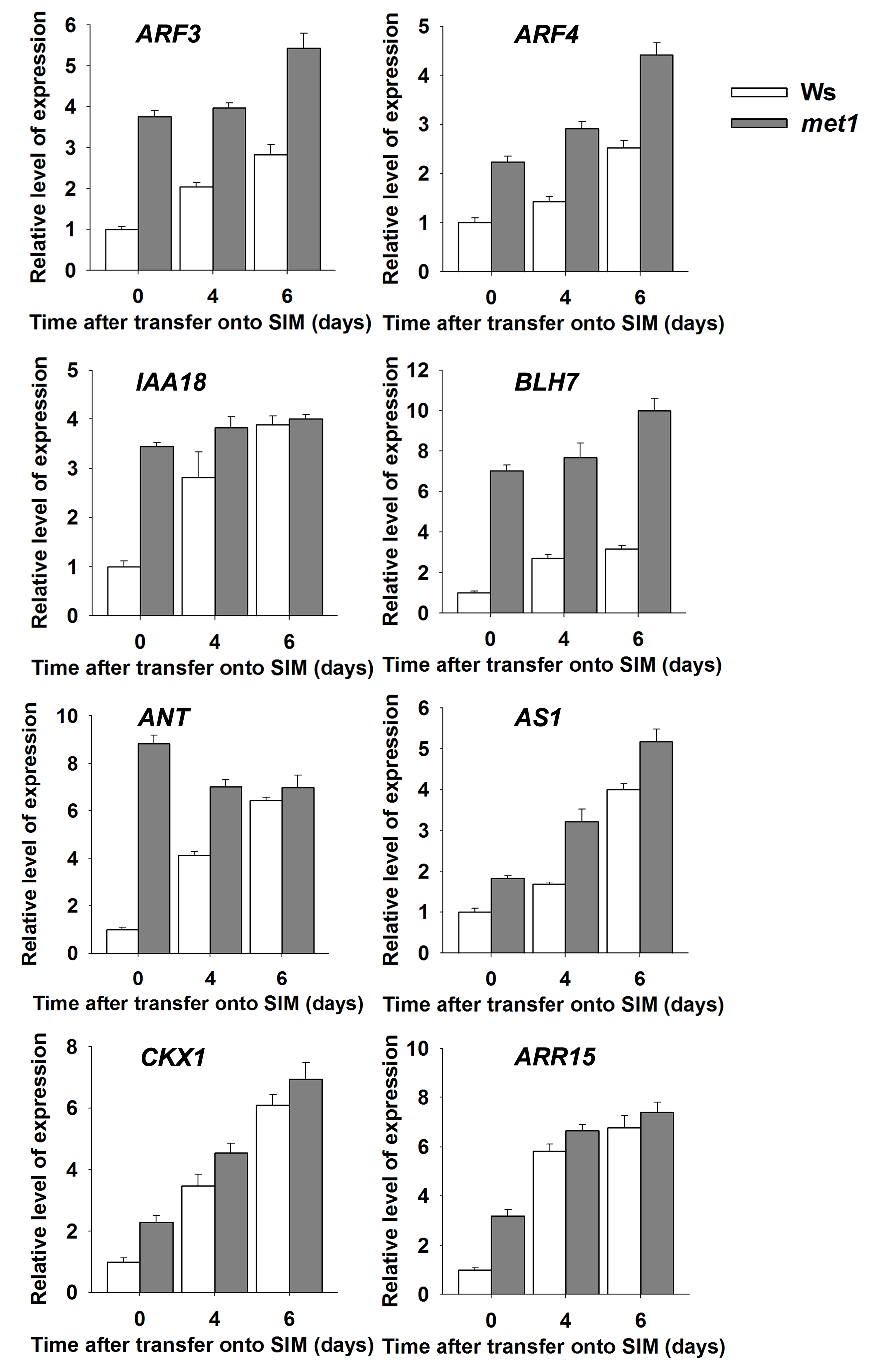

Supplement: Figure S4 — Expression patterns of candidate genes validated by qRT-PCR. Total RNAs were isolated from calli of wild type and met1 cultured on SIM at the indicated time points, and the transcripts of genes ARF3, ARF4, IAA18, BLH7, ANT, AS1, CKX1, and ARR15 were measured by qRT-PCR. Three independent RNA preparations were analyzed for each time point. Mean values were calculated from triplicate qRT-PCR analysis with standard errors. The relative expression level of each gene, corresponding to the expression level of TUBULIN2, was calculated using the comparative C(T) method. (TIF) [file pgen.1002243.s004.tif]
