# Supplementary material for: DNA Methylation and Histone Modifications Regulate De Novo Shoot Regeneration in Arabidopsis by Modulating WUSCHEL Expression and Auxin Signaling
Source: PLoS Genet. 2011 Aug 18;7(8):e1002243. doi: 10.1371/journal.pgen.1002243 (PMC3158056; doi:10.1371/journal.pgen.1002243)
Supplement: Table S1 — The percentage of the calli with WUS expressing signals detected by in situ hybridization. (DOC) [file pgen.1002243.s005.doc]

**Table S1.** The percentage of the calli with *WUS* signals detected by *in situ* hybridization.

| *Genotype* | *0 day (%)* | *2 days (%)* | *4 days (%)* | *6 days (%)* |
| --- | --- | --- | --- | --- |
| Ws | 0±0 | 0±0 | 60.40±2.31 | 80.21±1.86 |
| *met1* | 0±0 | 48.26±1.38 | 82.58±3.28 | 95.65±2.17 |
| L*er* | 0±0 | 0±0 | 36.96±3.40 | 55.88±2.55 |
| *kyp-2* | 0±0 | 27.58±2.26 | 51.21±1.70 | 84.01±2.25 |
