# Supplementary material for: DNA Methylation and Histone Modifications Regulate De Novo Shoot Regeneration in Arabidopsis by Modulating WUSCHEL Expression and Auxin Signaling
Source: PLoS Genet. 2011 Aug 18;7(8):e1002243. doi: 10.1371/journal.pgen.1002243 (PMC3158056; doi:10.1371/journal.pgen.1002243)
Supplement: Table S2 — The number of pWUS::GUS signal distribution detected in each callus. (DOC) [file pgen.1002243.s006.doc]

**Table S2.** The number of *pWUS::GUS* signal distribution detected in each callus.

| *Genotype* | *6 days* | *8 days* | *10 days* | *14 days* |
| --- | --- | --- | --- | --- |
| Ws | 0±0 | 0±0 | 3±1.0 | 10±1.5 |
| *met1* | 0±0 | 2±1.0 | 9±1.5 | 16±2.5 |
